# Endothelium Infection and Dysregulation by SARS-CoV-2: Evidence and Caveats in COVID-19

**DOI:** 10.3390/v13010029

**Published:** 2020-12-26

**Authors:** Isabelle Bernard, Daniel Limonta, Lara K. Mahal, Tom C. Hobman

**Affiliations:** 1 Department of Medical Microbiology & Immunology, University of Alberta, Edmonton, AB T6G 2E1, Canada; icbernar@ualberta.ca; 2 Department of Cell Biology, University of Alberta, Edmonton, AB T6G 2H7, Canada; dlimonta@ualberta.ca; 3 Li Ka Shing Institute of Virology, University of Alberta, Edmonton, AB T6G 2E1, Canada; 4 Department of Chemistry, University of Alberta, Edmonton, AB T6G 2G2, Canada; lkmahal@ualberta.ca; 5 Women & Children’s Health Research Institute, University of Alberta, Edmonton, AB T6G 1C9, Canada

**Keywords:** COVID-19, SARS-CoV-2, ACE2, RAAS, bradykinin–kallikrein pathway, ADAM17, endothelial dysfunction, pericyte, immunothrombosis, therapeutics

## Abstract

The ongoing pandemic of coronavirus disease 2019 (COVID-19) caused by the acute respiratory syndrome-coronavirus-2 (SARS-CoV-2) poses a persistent threat to global public health. Although primarily a respiratory illness, extrapulmonary manifestations of COVID-19 include gastrointestinal, cardiovascular, renal and neurological diseases. Recent studies suggest that dysfunction of the endothelium during COVID-19 may exacerbate these deleterious events by inciting inflammatory and microvascular thrombotic processes. Although controversial, there is evidence that SARS-CoV-2 may infect endothelial cells by binding to the angiotensin-converting enzyme 2 (ACE2) cellular receptor using the viral Spike protein. In this review, we explore current insights into the relationship between SARS-CoV-2 infection, endothelial dysfunction due to ACE2 downregulation, and deleterious pulmonary and extra-pulmonary immunothrombotic complications in severe COVID-19. We also discuss preclinical and clinical development of therapeutic agents targeting SARS-CoV-2-mediated endothelial dysfunction. Finally, we present evidence of SARS-CoV-2 replication in primary human lung and cardiac microvascular endothelial cells. Accordingly, in striving to understand the parameters that lead to severe disease in COVID-19 patients, it is important to consider how direct infection of endothelial cells by SARS-CoV-2 may contribute to this process.

## 1. Introduction

Coronavirus disease-2019 (COVID-19) is primarily a respiratory illness caused by the severe acute respiratory syndrome-coronavirus-2 (SARS-CoV-2). In December 2019, pneumonia cases of unknown etiology were reported in Wuhan, the capital city of Hubei province in China [1,2], after which the new coronavirus spread globally and was consequently deemed a pandemic by the World Health Organization in March 2020 [3]. 

COVID-19 symptoms most commonly reported include fever, cough, and shortness of breath or difficulty breathing [4,5]. In more severe cases, the disease can progress to acute respiratory distress syndrome (ARDS) and hypoxic respiratory failure, which is the leading cause of mortality in COVID-19 patients [4,5,6]. Although pulmonary manifestations are the major presentations of COVID-19, other important extrapulmonary events include gastrointestinal complications [7,8], cardiovascular injury [7,9], renal dysfunction [7,10,11], and neurological disorders [7,12,13]. Multiple studies have found that microvascular thrombotic and inflammatory processes may play a role in exacerbating ARDS and extrapulmonary events in COVID-19 patients [10,14,15,16]. These deleterious complications likely result from dysfunction of the vascular endothelium [10,15,16,17]. 

**Figure 1:**
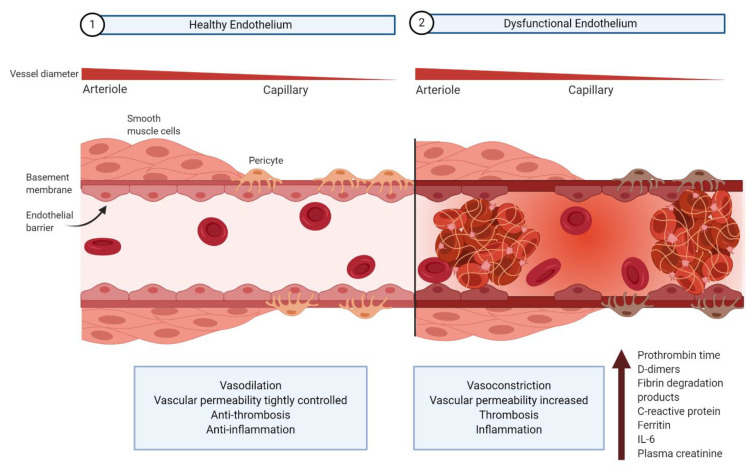
Architecture of healthy and dysfunctional vascular endothelium. (1) In homeostatic conditions, a healthy endothelium is a thin monolayer of endothelial cells at the interface between the circulation and tissue. A basement membrane separates the endothelium from smooth muscle and underlying connective tissue in arterioles. Smooth muscle cells regulate vascular tone, promoting vasodilation in conjunction with endothelial cells. As the vessel diameter decreases in capillaries, the vessel walls consist uniquely of the endothelial monolayer, basement membrane, and pericytes within the basement membrane wrapping around the abluminal surface of endothelial cells. Functionally, pericyte–endothelial cell cross-talk is essential to maintain normal endothelial cell shape and function. Together, endothelial cells, smooth muscle cells, and pericytes promote vasodilation, limit vascular permeability, promote an anti-thrombotic state, and reduce hyperinflammation in healthy patients. (2) However, disruption of the endothelial barrier and endothelial cell function leads to deleterious vasoconstriction, increased vascular permeability, thrombosis, and hyperinflammation. Dysfunction of the endothelium is indicated by a variety of biomarkers.

The vascular endothelium, a monolayer of endothelial cells lining the inner wall of blood and lymph vessels, maintains homeostasis by regulating systemic blood flow and tissue perfusion in conjunction with underlying smooth muscle cells and pericytes (Figure 1) [18,19,20,21]. In healthy individuals, endothelial cells promote vasodilation, regulate vascular permeability, promote an anti-thrombotic state, and regulate the immune response [18,19,20]. Endothelial dysregulation accordingly results in vasoconstriction, vascular leakage, thrombosis, hyperinflammation, and dysregulation of the antiviral immune response [18,19,20].

Abnormal coagulation and inflammation parameters provide evidence for endothelium dysregulation in severe COVID-19 patients. Prolonged clot formation indicated by an elevated prothrombin time, as well as increased D-dimers, fibrin degradation products, and ferritin levels are all correlated with hypercoagulation [16,22,23,24]. Deleterious inflammation is indicated clinically by an elevation in C-reactive protein and interleukin-6, both of which are associated with increased disease severity [16,22,24]. Elevated plasma creatinine is another indication of endothelial barrier dysfunction, related to the kidney filtering function, in COVID-19 patients [24,25]. 

Endothelial dysfunction is also a common denominator in reported COVID-19 comorbidities [10]. A case series of 5700 hospitalized COVID-19 patients reported that hypertension, obesity, and diabetes are the most common comorbidities [26], all of which involve underlying endothelial damage [10]. Together, major clinical events in severe COVID-19, laboratory evidence of endothelial dysfunction associated with poor prognosis, and reported comorbidities all support that COVID-19 targets endothelial cells [10,15]. The pathophysiological mechanisms underlying endothelial dysfunction in COVID-19 still require further clarification. Following entry of SARS-CoV-2 in the upper and lower respiratory tract, viral infection likely occurs first in airway epithelial cells, vascular endothelial cells, and lung macrophages [27]. SARS-CoV-2 may thus cause endothelial dysfunction either directly through endothelial cell infection, or indirectly through the infection of other susceptible cell types, which cause hyperinflammation and aberrant antiviral responses [14,17,27]. Endothelial damage is an important culprit in multi-organ immunothrombosis and contributes to severe COVID-19 disease progression [10,14,15,16,28].

Despite promising efforts to develop therapies targeting COVID-19-mediated endothelial dysfunction, COVID-19 patient management as recommended by the National Institute of Health (NIH) is currently limited to the disputed antiviral agent remdesivir, a nucleotide analogue [29,30,31,32,33], and the corticosteroid dexamethasone [29,34]. This highlights the need to develop effective therapies targeting COVID-19-mediated endothelial dysfunction. This review discusses current insights into the relationship between SARS-CoV-2 and endothelial dysfunction to provide a better mechanistic understanding of pulmonary and extra-pulmonary manifestations of COVID-19. We will also discuss investigational therapeutic strategies proposed for SARS-CoV-2-mediated endothelial dysfunction based on preclinical and clinical findings. 

## 2. Search Strategy

The references used in this review were identified through searches on PubMed, Ovid MEDLINE, Google Scholar, bioRxiv/medRxiv, and ClinicalTrials.gov from 28 June 2020 to 13 December 2020. The search strategy was developed by combining relevant terms in endothelium and endothelial dysfunction related to 2019-nCoV, COVID-19, and SARS-CoV-2 (Supplementary Material S1). From a search strategy combining these terms, 3998 papers were published in PubMed, 4492 in Ovid MEDLINE, 13,200–16,300 in Google Scholar, 11,209 in bioRxiv/medRxiv, and 87 in ClinicalTrials.gov. Subsequent searches on specific topics related to endothelial infection and dysregulation in COVID-19 were done using this search strategy.

## 3. Entry Receptors and Tropism of SARS-CoV-2 and Related Human Coronaviruses

SARS-CoV-2 belongs to the family *Coronaviridae*, a group of large enveloped positive-sense single-stranded RNA viruses that is divided into five genera [35]. They cause a broad spectrum of animal and human diseases, although pulmonary disease is most characteristic in humans infected with alphacoronaviruses (229E, NL63) and betacoronaviruses (OC43, HKU1, MERS-CoV, SARS-CoV-1, SARS-CoV-2) [2,35]. Sequence analysis of the SARS-CoV-2 genome indicates that it shares 79.6% nucleotide identity with the related SARS-CoV-1, the viral agent responsible for the 2003 SARS pandemic [2]. Previous research on the pathogenesis and treatment of SARS-CoV-1 infection has thus guided the ongoing investigation of SARS-CoV-2 in many aspects. 

The pathogenesis of coronaviruses depends largely on the mechanism of entry and subsequent actions in target cells. Entry is mediated by the viral spike (S) glycoprotein, which can be post-translationally cleaved into an N-terminal receptor-binding subunit (S1) and membrane-anchored subunit (S2) before binding to a receptor on the target cell followed by virus-cell fusion [36]. Prior to 2003, the cell surface metalloprotease aminopeptidase N (APN, CD13) was identified as the receptor for the human alphacoronavirus 229E [37]. Human betacoronaviruses OC43 and HKU1 use *O*-acetylated sialic acids (*O*-Ac-SA) as cellular attachment factors [38,39], while MERS used dipeptidyl peptidase 4 (DPP4) [40]. Of note, Li et al. found that a metallopeptidase, angiotensin-converting enzyme 2 (ACE2), was the receptor for SARS-CoV-1 [41]. Furthermore, expression of ACE2 in cells not previously susceptible to SARS-CoV-1 infection facilitated viral entry, highlighting ACE2 as a necessary and sufficient functional receptor for the virus [41]. Subsequent studies confirmed that ACE2 was also the receptor for the alphacoronavirus NL63 [42].

Investigation into tissue and cellular distribution of ACE2 revealed that ACE2 expression correlates well with tissue tropism of SARS-CoV-1 [43,44,45]. Abundant expression of ACE2 in endothelial cells, immune cells, and epithelial cells of the lung, intestine, heart and kidney [45,46], together with evidence of SARS-CoV-1 particles in these tissues are also consistent with these observations [43,47,48,49]. However, an in-situ hybridization analyses of tissues from fatal SARS cases found no viral particles in vascular endothelial cells despite the fact that these cells express relatively high levels of ACE2 [43,45,47]. Such discrepancies between tissue distribution of ACE2 and SARS-CoV-1 tropism may indicate that the virus uses different receptors or a distinct set of co-receptors in different organs [43,44,45]. For example, the cellular transmembrane protease serine 2 (TMPRSS2) primes the S-protein and activates virus-plasma membrane fusion, thus affecting tropism [50,51]. Further studies should elucidate whether other host receptors are involved in the tissue-specific tropism of SARS-CoV-1.

Recently, Hoffman et al. were able to show that SARS-CoV-2 also uses ACE2 for entry and TMPRSS2 for S-protein priming [52]. Subsequent crystallographic and cryo-electron microscopy analyses of the homotrimeric S protein structure confirmed its functional interaction with ACE2 [53,54,55,56,57]. Turoňová et al. [58] then used cryo-electron tomography, subtomogram averaging, and molecular dynamics simulations to show that the S-protein contains hinges protected by glycosylation sites, thus conferring flexibility that may allow it to scan the host cell surface and efficiently bind ACE2. Interestingly, analyses of the crystal structure of the S1 protein receptor binding domain (RBD) indicate that SARS-CoV-2 RBD has higher affinity for ACE2 than SARS-CoV-1 RBD, which is consistent with more efficient cell entry [53,59]. 

Similar to what was observed with SARS-CoV-1, abundant expression of ACE2 and TMPRSS2 generally correlates with SARS-CoV-2 tropism [60,61,62]. However, recent studies have found discrepancies between SARS-CoV-2 tropism and cellular distribution of ACE2 and TMPRSS2. For example, human colorectal adenocarcinoma cell lines HT29 and DLD1 express both ACE2 and TMPRSS2, yet are not permissive to SARS-CoV-2 replication in vitro [63]. In contrast, the colon adenocarcinoma cell line CL14 is permissive to SARS-CoV-2 although it expresses lower levels of ACE2 than HT29 and DLD1 [63]. These findings suggest that SARS-CoV-2 may rely on different sets of co-receptors depending upon cell type.

## 4. Does SARS-CoV-2 Infect Endothelial Cells?

Whether SARS-CoV-2 directly infects endothelial cells remains a matter of controversy. In addition to ACE2 and TMRPSS2, endothelial cells express other factors involved in viral entry of SARS-CoV-1 and SARS-CoV-2. As well as being sufficient to promote SARS-CoV-1 S-protein-mediated membrane fusion, the endosomal cysteine proteases, cathepsin B and L, have been shown to enhance SARS-CoV-2 entry by S-protein priming in the absence of TMPRSS2 [64,65]. Heparan sulfate, a highly charged glycosaminoglycan, has also been shown to enhance binding [66]. Recent structural analyses additionally suggest that SARS-CoV-2 binds to sialic acid-containing glycoproteins and gangliosides on endothelial cells [67]. CD147 may serve as a viral attachment factor for SARS-CoV-2 entry on the basal surface of the endothelium, although its significance in viral entry remains unclear [68,69]. Finally, unlike SARS-CoV-1 virions which do not undergo cleavage by the proprotein convertase furin (expressed in many cell types including endothelial cells), SARS-CoV-2 particles are reportedly pre-activated by this protease during entry into host cells [59]. Proteolytic cleavage of polybasic furin-type cleavage sites in the SARS-CoV-2 Spike protein was recently shown to expose a conserved C-terminal motif that binds cell surface Neuropilin-1 and Neuropilin-2 receptors, thus significantly potentiating SARS-CoV-2 entry and infectivity in vitro [70,71]. As pulmonary and olfactory endothelial cells abundantly express Neuropilin-1 and Neuropilin-2 [70], furin-mediated cleavage of the SARS-CoV-2 Spike receptor may potentiate efficient cell entry and infectivity in the endothelium. Together, the expression of ACE2, TMPRSS2, and other entry factors in endothelial cells is at least consistent with a scenario in which these cells can be directly infected by SARS-CoV-2. 

Using electron microscopy (EM) analyses of post-mortem samples, Varga et al. reported evidence of viral inclusion bodies in the peritubular space and viral particles in renal endothelial cells of one of the three COVID-19 patients presenting with endotheliitis [72]. Several other EM studies also noted viral-like particles [14,73,74,75] in endothelial cells. Ackermann et al. [14] reported intracellular and extracellular SARS-CoV-2 virions in endothelial cells of the lung microvasculature from one of seven patients who died from COVID-19. Also using EM, Paniz-Mondolfi et al. described transcellular migration of viral-like particles from brain microvascular endothelial cells to their neural niche in a postmortem COVID-19 patient sample [73]. Interestingly, a study investigating the endothelium of fourteen COVID-19 patients post-mortem documented SARS-CoV-2-like particles in renal endothelial cells, although these were not observed in other surveyed organs [75]. Detection of SARS-CoV-2 in endothelial cells is further supported by Colmenero et al., who retrospectively examined skin biopsies from seven pediatric COVID-19 patients presenting with chilblains [74]. They reported cytoplasmic granular positivity for the S-protein in the vascular endothelium through immunohistochemical analysis [74], which was corroborated by another study indicating the presence of SARS-CoV-2 protein in renal endothelial cells [76]. Structurally similar viral-like particles and tubular-reticular inclusions were also reported in endothelial cells of foot and toe biopsy specimens from these pediatric chilblains patients, which is consistent with previous characterization of SARS-CoV-1 infection and may indicate an enhanced type I interferon response against viral attack [74,77,78,79]. 

In contrast to the those cited above, a recent study found no evidence of the virus in the vascular endothelium after immunohistochemical staining of ex vivo lung cultures from one patient infected with SARS-CoV-2 [80]. This finding is supported by a report that failed to detect immunohistochemical reactivity in post-mortem pulmonary endothelial cells of five COVID-19 patients with diffuse alveolar damage [81]. Another group also reported that endothelial cells derived from human pluripotent stem cells are largely resistant to infection with SARS-CoV-2 [82] and multiple studies challenge the characterization of structures seen under EM as coronavirus particles. For example, Goldsmith et al. proposed that the EM images described by Varga et al. [72] could be cross-sections of the rough endoplasmic reticulum, where ribosomes have been mistaken for coronavirus spikes [83]. A report investigating putative virions by EM of three biopsies from live COVID-19 patients found similar structures to those reported in the literature [14,73,74,75], although these structures were re-characterized as clathrin-coated vesicles and multivesicular bodies intracellularly, or extruded microvesicles and degenerate microvilli if extracellular [84]. Several other reports similarly challenge the identification of “viral-like particles” and instead suggest these structures may be vesicles or subcellular multivesicular bodies with associated structures that resemble viral spikes [84,85,86,87]. In fact, in a correspondence by Dittmayer et al. [87], the reported identification of SARS-CoV-2 particles in renal endothelial cells [75] is challenged, amongst several other putative EM reports of intracellular viral particles. The authors did present evidence of SARS-CoV-2 particles in endothelial cells from a post-mortem lung specimen of one COVID-19 patient, importantly also confirming high SARS-CoV-2 RNA load in this specimen using RT-PCR [87]. Therefore, although EM remains a powerful method for detecting the presence of virions in cells, SARS-CoV-2 diagnosis in cells should be carefully combined with concurrent detection of viral proteins or nucleic acids to prevent misleading characterization of viral particles [84,87]. 

Despite the controversy over EM studies of infected endothelial cells, SARS-CoV-2 virions in bronchial epithelial cells [88] were reportedly similar to the “viral” particles described in endothelial cells by the aforementioned studies [72,73,74,75,89]. Furthermore, strikingly similar viral-like particles in other cell types known to be permissive to SARS-CoV-2 infection [90,91] have been described; viral-like SARS-CoV-2 particles were reported in type II alveolar epithelial cells in one COVID-19 patient [92], in renal podocytes of one COVID-19 patient with acute kidney injury [93], and in the post-mortem tubular epithelium of nine COVID-19 patients with acute kidney injury from 26 surveyed [94]. Limited expression of SARS-CoV-2 was also detected in the maternal decidua parietalis endothelium in two of 19 COVID-19-positive pregnant women through immunohistochemistry and/or in situ RNA hybridization [95]. Skin biopsies of acral perniotic lesions surveyed during the pandemic found immunohistochemical evidence of the SARS-CoV-2 S-protein in three of the six patients, but no evidence of viral RNA through in-situ RNA hybridization [96]. Nevertheless, further studies in clinical samples—particularly immuno-EM and in situ RNA analysis—are required to definitively ascertain whether SARS-CoV-2 can productively infect endothelial cells. Regardless of whether SARS-CoV-2 directly infects endothelial cells, there is no question that multi-organ endothelial dysfunction is a characteristic of severe COVID-19 [10,15,17,97]. 

**Figure 2:**
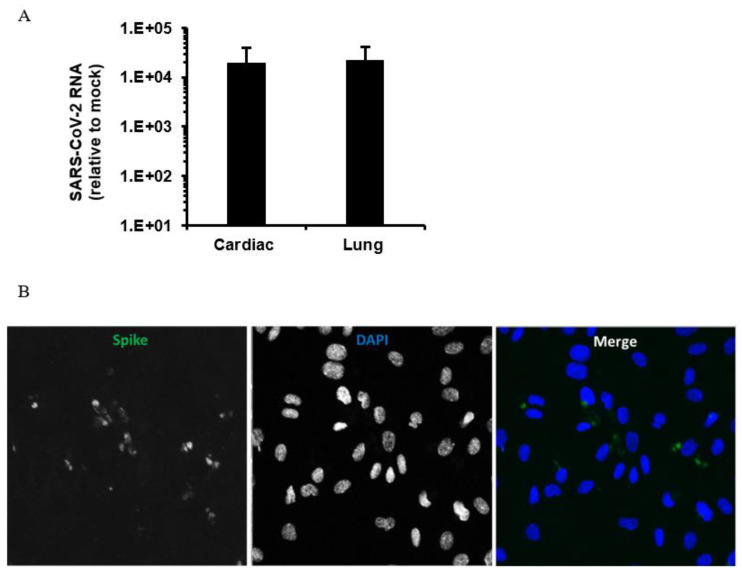
SARS-CoV-2 infects human endothelial cells. (**A**) Human primary lung and cardiac microvascular cells were infected with SARS-CoV-2 (MOI = 3) for 48 h followed by cell lysates collection for RNA isolation and qRT-PCR using SYBR Green (Quanta BioSciences). Viral spike protein expression was quantitated using a Bio-Rad CFX96 Touch System. Values are expressed as the mean of three independent experiments. Error bars represent standard errors of the mean. (**B**) Representative confocal imaging (20×) of human primary lung microvascular cells infected with SARS-CoV-2 (MOI = 3) for 48 h. After fixation, coronavirus-infected cells were stained using mouse monoclonal antibody to SARS spike protein (GeneTex) and Alexa Fluor 488 donkey anti-mouse (Invitrogen). Nuclei were stained with DAPI. Confocal images were acquired using Olympus 1 × 81 spinning disk confocal microscope with Volocity 6.2.1 software.

To address the potential infection of endothelial cells by SARS-CoV-2, we infected human microvascular endothelial cells (Lonza) from lung and heart with a Canadian isolate of SARS-CoV-2 (SARS-CoV-2/CANADA/VIDO 01/2020). A multiplicity of infection of 3 was used with 1 h of adsorption followed by incubation for 48 h followed by extraction of total cell RNA (Macherey-Nagel GmbH & Co., Düren, Germany) and collection of media for virus RNA quantitation by qRT-PCR [2] and viral titer determination by plaque assay in Vero E6 cells respectively. Lung endothelial cells on coverslips were also infected, using the same conditions before processing for indirect immunofluorescence and confocal microcopy analysis as described [98]. We observed very low titers (3–4.5 10^2 PFU/mL) together with modest viral RNA levels in infected endothelial cells (Figure 2A). Confocal imaging of infected endothelial cells confirmed expression of the SARS-CoV-2 spike protein in a proportion of the cells (Figure 2B). These data indicate that human endothelial cells are moderately permissive for infection with SARS-CoV-2. Conversely, using a SARS-CoV-2-GFP reporter virus (MOI = 0.5–3 with 2 h of adsorption) and human primary lung microvascular endothelial cells generated in-house (2 donors), Hou et al. [99] found no GFP signal in cells or detectable infectious titers in supernatants after 48 h of infection. 

## 5. Cellular ACE2 Downregulation Leading to Endothelial Dysfunction Is Mediated by SARS-CoV-2

In this section, we discuss how SARS-CoV-2 infection may damage the endothelium upon binding to ACE2 on endothelial cells and other susceptible cells. Following attachment to ACE2, SARS-CoV-2 is internalized into susceptible cells and concurrently downregulates this receptor, a phenomenon that is associated with dysfunction of the Renin-Angiotensin-Aldosterone System (RAAS) and the bradykinin–kallikrein pathway. Cellular ACE2 downregulation may also result from ADAM17-mediated shedding, although the pathologic implications of this process are unclear. 

### 5.1. RAAS Dysfunction

**Figure 3:**
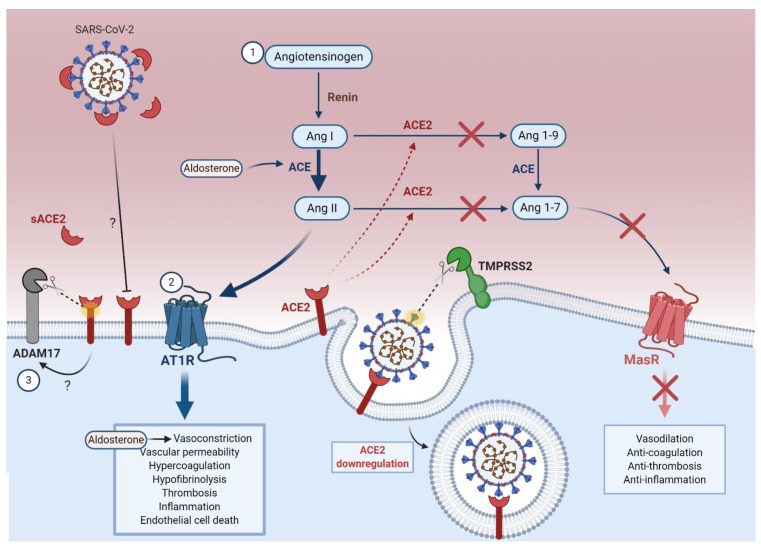
SARS-CoV-2-mediated cellular ACE2 downregulation leads to RAAS dysfunctions. (1) In normal conditions, the liver releases angiotensinogen, which is hydrolyzed into Ang I by renin (angiotensinogenase) from renal juxtaglomerular cells. ACE produced in the kidneys and lungs further converts Ang I into Ang II, which are then converted by ACE2 into Ang 1-9 and Ang 1-7 respectively. Ang 1-7 binds to MasR to mediate vasoprotective effects. (2) During SARS-CoV-2 infection, viral internalization results in cellular ACE2 downregulation and increased ACE. Consequently, Ang II accumulates and binds to AT1R, mediating detrimental vascular effects. (3) ADAM17 proteolytically sheds sACE2, which exacerbates inflammation, may further amplify RAAS dysfunction by decreasing cellular ACE2, and blocks SARS-CoV-2 entry.

The RAAS is a complex hormonal network that regulates hemodynamic stability [100] (Figure 3). In response to renal artery hypotension, renin (angiotensinogenase) released from juxtaglomerular cells hydrolyzes angiotensinogen into angiotensin-I (Ang I) [100]. Angiotensin converting enzyme (ACE) converts Ang I into angiotensin-II (Ang II), which binds to Ang II receptor type 1 (AT1R) on endothelial cells [100,101]. Ang II promotes microvascular thrombosis [102], coagulopathy [103,104], hypofibrinolysis (reduced breakdown of fibrin in blood clots) [104] and pro-inflammatory processes [102,103], while exerting powerful vasoconstrictive effects through the hormone aldosterone [97,100,101]. ACE2 catalyzes the conversion of Ang II into angiotensin-(1-7) (Ang 1-7), which binds to the cellular Mas receptor to counteract the effects of Ang II [105,106,107]. Interestingly, the vascular endothelium has been shown to contribute to both ACE-2-dependent and -independent generation of Ang 1-7, therefore being essential to maintain homeostasis [108].

Studies on the role of ACE2 in SARS pathogenesis have facilitated our understanding of its potential role in COVID-19 pathogenesis. SARS-CoV-1 S-protein binding to ACE2 was shown to downregulate expression of ACE2 in vitro and in vivo [109,110]. ACE2 downregulation is associated with poor prognosis in SARS and COVID-19 patients, by exacerbating ARDS [111,112,113], severe pneumonia [110,112], and extra-pulmonary conditions including acute kidney injury and cardiac injury [111,112]. Lung failure due to injection of SARS-CoV-1 S-protein into mice could be partially reversed by blocking the RAAS in vivo [110]. Ferrario et al. also demonstrated that upregulation of ACE2 counterbalances the pathological upregulation of the RAAS in cardiac cells [114]. Together, these results are consistent with a scenario in which downregulation of ACE2 in SARS-CoV-2-susceptible cells (including endothelial cells) leads to increased pathological activity of the RAAS, which further accelerates the progression of SARS, and very likely COVID-19, from a mild to severe diseases.

#### 5.1.1. ACE2 Downregulation Increases the Ang II to Ang 1-7 Ratio

The downregulation of ACE2 following coronavirus infection impairs the conversion of Ang I to Ang 1-9 and Ang II to Ang 1-7, resulting in the intracellular accumulation of Ang II [105,106] (Figure 3). Moreover, an early investigation of epidemiological, clinical, laboratory, and radiological markers of COVID-19 disease severity in China found that elevated serum Ang II was positively correlated with viral load and lung injury in COVID-19 [115], likely due to AT1R activation as well as endothelial cell death by oxidative stress and ubiquitin-proteasome-mediated proteolysis [116,117,118]. Whether AT1R upregulation occurs in COVID-19 or contributes to hypertensive complications requires further investigation. 

#### 5.1.2. Ang II Upregulation Increases the PAI-1 to tPA/uPA Ratio

**Figure 4:**
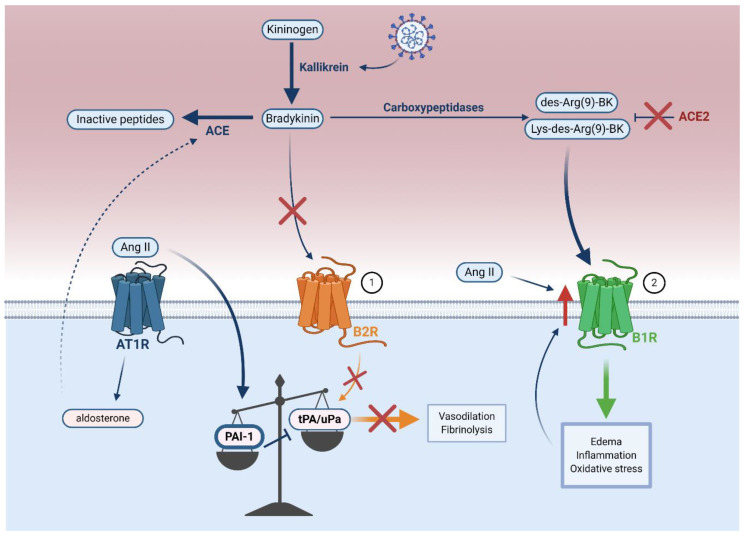
SARS-CoV-2-mediated cellular ACE2 downregulation leads to bradykinin–kallikrein pathway dysfunction. (1) In normal conditions, kallikrein converts kininogen to bradykinin, which can then bind to B2R on endothelial cells. B2R stimulation increase tPA/uPA to mediate protective vasodilation and fibrinolysis. (2) During SARS-CoV-2 infection, increased activity of kallikrein increase bradykinin. However, ACE2 downregulation increases Ang II. Ang II can convert bradykinin to its inactive peptides through aldosterone-induced ACE expression and increase PAI-1, which inhibits the protective effects of tPA/uPA. Furthermore, ACE2 downregulation prevents the inactivation of des-Arg(9)-BK and Lys-des-Arg(9)-BK. These can bind to endothelial B1R to promote detrimental vascular effects, which together with Ang II, upregulate B1R. In all, inhibition of B2R and stimulation of B1R results in hypofibrinolysis and adverse vascular consequences.

Accumulation of Ang II promotes thrombosis by inducing the expression of plasminogen activator inhibitor-1 (PAI-1) in endothelial cells [102,104,119,120] (Figure 4). PAI-1 inhibits tissue plasminogen activator (tPA) and urokinase plasminogen activator (uPA), two proteins that mediate fibrinolysis [97]. The increased PAI-1 to tPA/uPA ratio observed in COVID-19 results in hypofibrinolysis, which likely leads to vascular microthrombosis and unresolved fibrin deposits in the alveoli [121,122].

In COVID-19 patients, the bradykinin–kallikrein pathway may counteract the effects of Ang II on the PAI-1 to tPA/uPA ratio in controlling acute inflammation by increasing the activity of kallikrein, a protease that converts kininogen to bradykinin [123,124]. Bradykinin binds to the bradykinin receptor B2 (B2R) constitutively expressed on endothelial cells [123,125] and is also upregulated in COVID-19 [124]. It is known that this binding stimulates release of tPA in the human vasculature, which is associated with fibrinolysis and vasodilation [124,126]. A more recent clinical study of 118 hospitalized COVID-19 patients reported elevated plasma tPA and PAI-1, in which high tPA levels strongly correlated with mortality by promoting spontaneous fibrinolysis [127]. The proposed mechanism is that endothelial cell infection and destruction potentiate deleterious tPA release, leading to hyperfibrinolytic bleeding complications in a subset of COVID-19 patients [127,128,129]. Nonetheless, in most patients, increased tPA is likely not sufficient to counterbalance the systemic pathological effects of PAI-1 during COVID-19 [97,107]. 

ACE mediates the breakdown of bradykinin to its inactive peptides [130] (Figure 4) and increased aldosterone-induced ACE expression in COVID-19 (Figure 3) may inactivate bradykinin, consequently preventing bradykinin-mediated increase in tPA [130,131]. Overall, it seems that an increased PAI-1 to tPA/uPA ratio leads to hypofibrinolysis in COVID-19. 

### 5.2. Bradykinin–Kallikrein Pathway Dysfunction

In addition to ACE-mediated decrease of tPA, ACE2 can directly control the actions of bradykinin. By injecting Ang II into ex vivo lungs of *Ace2* knockout mice, Imai et al. found that pulmonary edema correlated with reduced ACE2 expression, but this was not due to Ang II-mediated hemodynamic alterations [132]. It is therefore likely that in COVID-19, ACE2 not only mediates pathological RAAS activity, but also facilitates deleterious bradykinin pathway activity independently of RAAS [123,132,133].

Bradykinin is further processed into des-Arg(9)-BK and Lys-des-Arg(9)-BK by carboxypeptidases [133,134] (Figure 4). Under normal conditions, ACE2 protects against pulmonary edema by inactivating des-Arg(9)-BK and Lys-des-Arg(9)-BK [134]. ACE2 depletion would likely block the inactivation of these two kinins, which would then be free to activate the endothelial bradykinin receptor B1 (B1R) and promote edema, inflammation, and oxidative stress in COVID-19 [133,134]. Further investigation of serum bradykinin and B1R levels in COVID-19 patients remains crucial to confirm whether B1R-mediated dysregulation of the bradykinin–kallikrein pathway occurs in COVID-19.

### 5.3. ADAM17-Mediated ACE2 Shedding

As well as internalization of ACE2 following SARS-CoV-2 infection, downregulation of this receptor can occur when ACE2-coronavirus complexes are shed from endothelial cells or other susceptible cell types [135]. Lambert et al. reported that ACE2 undergoes ADAM metallopeptidase domain 17 (ADAM17)-mediated proteolytic shedding shortly after binding to the SARS-CoV-1 S-protein [135] (Figure 3). This has two major implications: first, further downregulation of membrane-bound ACE2 by ADAM17 amplifies RAAS and bradykinin–kallikrein-mediated pathology; and second, bioactive soluble ACE2 (sACE2) shed from endothelial cells can spread in the circulation and cause systemic inflammation [135,136,137,138,139].

ADAM17-mediated sACE2 shedding may also play a role in SARS-CoV-2 entry. A recent study reported increased mRNA expression of ADAM17 in alveolar epithelial cells in vitro following SARS-CoV-2 infection, although the implications in SARS-CoV-2 entry remained ambiguous [139]. Haga et al. found that SARS-CoV-1 infection was significantly reduced when ADAM17 expression was knocked down by siRNAs [138]. Intriguingly, they also found that the modulation of ADAM17 activity by SARS-CoV-1 requires the ACE2 cytoplasmic tail domain, and deleting this domain reduced SARS-CoV-1 infection [138]. Based on these results, the authors concluded that ADAM17 activity contributes to viral entry [138]. However, other studies did not find evidence supporting the role of ADAM17 in SARS-CoV-1 entry [136,140]. 

In contrast to previous SARS findings [138], several reports propose that sACE2 may actually have a protective effect against SARS-CoV-2 infection [141,142,143]. Monteil et al. [141] showed by RT-qPCR that clinical-grade human recombinant soluble ACE2 (hrsACE2) reduced SARS-CoV-2 replication by 1000–5000-fold in cell culture, engineered human blood vessels, and kidney organoids. As evidence of its clinical efficacy, Zoufaly et al. [142] presented a case report of hrsACE2 first-course treatment in a patient with severe COVID-19. A marked reduction in inflammatory markers and Ang II, along with a concomitant increase in Ang 1-7 and Ang 1-9, were reported after administration of hrsACE2 [142]. Importantly, SARS-CoV-2-specific RT-PCR showed rapid viral clearance until 12 days post-treatment [142]. It is believed that by binding the SARS-CoV-2 S-protein, sACE2 prevents its association with membrane-bound ACE2 and effectively blocks viral internalization [141]—a mechanism that previously demonstrated in SARS-CoV-1 [41]. In fact, in a collaborative study with our group, Glasgow et al. [143] showed by RT-qPCR that highly optimized sACE2 was able to reduce replication of SARS-CoV-2 in Vero E6 cells more than 50,000-fold. Emerging reports of sACE2 neutralization capacity in COVID-19 are promising, although further research is required to elucidate its therapeutic efficacy. 

## 6. Consequences of Endothelium Dysfunction in COVID-19

In this section, we discuss how SARS-CoV-2-mediated endothelium dysfunction contributes to pathology in severe COVID-19, either directly through productive infection, or indirectly through immune mechanisms caused by infection of other susceptible cells. Furthermore, we discuss how this impacts the disease severity. 

### 6.1. Dysfunction of Pericyte–Endothelial Cell Cross-Talk

As discussed, SARS-CoV-2-mediated downregulation of ACE2 may increase the permeability of the endothelium via the RAAS and bradykinin–kallikrein pathway [115,124,134,144]. A leaky endothelial junction may allow the movement of SARS-CoV-2 virions from the microcirculation to pericytes—cells in the basement membrane that surround the abluminal surface of the endothelial cells (Figure 1) [145,146,147]. Recent studies have indicated that ACE2 is highly expressed in microvascular pericytes, making them targets for SARS-CoV-2 infection [147,148]. Although EM and immunohistochemical evidence validating the presence of SARS-CoV-2 particles in pericytes is so far lacking, post-mortem histological analysis of lung biopsies from two COVID-19 patients reveal critical pericyte loss or detachment [146]. These results are consistent with a scenario in which direct infection of microvascular pericytes leads to dysfunction of these cells. In fact, productive pericyte infection by the human immunodeficiency virus (HIV) and simian immunodeficiency virus (SIV) has been demonstrated in pericytes, and is believed to cause vascular abnormalities that characterize chronic HIV lung diseases [149]. 

Pericytes “cross-talk” with endothelial cells through a variety of mediators including platelet-derived growth factor receptor-β (PDGFR-β) and angiopoietin I (Angpt I) on pericytes with PDGF-B and Angpt II on endothelial cells [21,145,150,151]. The loss of pericytes following SARS-CoV-2 infection likely results in decreased PDGFR-β and Angpt I levels, which are correlated with abnormal endothelial cell shape, increased endothelial permeability, and thrombosis [21,145,150,151]. Pericyte ablation due to SARS-CoV-2 infection may thus contribute to endothelium-mediated thrombogenic complication observed in severe COVID-19 [145].

### 6.2. Immunothrombosis

Microvascular thrombosis is characteristic of severe COVID-19 [10,14,15,16,28] and it has been proposed that a virus-induced prothrombotic state culminates with hyperinflammatory effectors and platelets to form immunothrombotic clots [152,153]. Reports of microangiopathic complications in severe COVID-19—including disseminated intravascular coagulation (DIC), venous thromboembolism, and pulmonary embolism—support a role for immunothrombosis in viral pathogenesis [22,23,154]. 

#### 6.2.1. Hyperinflammation and Complement Activation

In early infection, SARS-CoV-2 delays the type I interferon (IFN) response through expression of the viral open reading frame 3b and open reading frame 6 proteins, which likely facilitates rapid viral replication in endothelial cells and other susceptible cell types at early stages of COVID-19 [155,156,157]. NFkB can subsequently drive a delayed and highly impaired type I IFN response associated with persistent viral load in the blood [155]. Together with significant release of pro-inflammatory cytokines TNF-α and IL-6 from endothelial cells in response to direct or indirect SARS-CoV-2-mediated endothelial injury, IFN deficiency and viral persistence lead to an exacerbated hyperinflammatory response [155]. 

SARS-CoV-2 was recently found to promote pro-inflammatory complement activation in association with endothelial damage in severe COVID-19 [152,153,158,159]. In fact, a study of 65 critically ill COVID-19 patients found that those who developed thromboembolic complications had significantly higher levels of mannose-binding lectin, which was specifically associated with complement pathway activation [159]. The SARS-CoV-2 N protein was found to potentiate complement lectin pathway activation [160], and Yu et al. also showed that the S protein activates the alternative complement pathway by binding heparan sulfate on cell surfaces [161]. 

In COVID-19, these activated pathways converge, resulting in production of complement anaphylatoxins C3a and C5a, which are known to increase immune cell recruitment, exacerbate endothelial damage by promoting release of reactive oxygen species, and exert deleterious pro-thrombotic effects [152,162,163]. Evidence for C3a/C5a production can be found in multiple clinical studies [158,163,164], one of which identified elevated plasma levels of C3a and C5a in 39 COVID-19 patients receiving maintenance hemodialysis [157]. Furthermore, treatment of COVID-19 patients with an anti-C5a antibody decreased systemic inflammation and increased lung oxygenation [160]. This further highlights the role of C3a/C5a complement activation in severe COVID-19. The pathophysiological mechanism of C3a/C5a in COVID-19 remains ambiguous, but these complement anaphylatoxins are known to stimulate inflammatory cytokine (notably IL-6 and TNF) release from macrophages and other cells expressing C3a/C5a receptors [165]. Heparan sulfate binding through alternative complement pathway activation also allows complement interaction with antithrombin III, which could exacerbate hypercoagulability in COVID-19, and various endothelial factors that could cause endothelial damage [161].

Increased formation of the cytolytic terminal soluble C5b-9 complex associated with respiratory failure was reported in a prospective cohort study of 39 hospitalized COVID-19 patients [166]. Apart from increasing recruitment and activation of neutrophils and monocytes that subsequently release pro-inflammatory cytokines, soluble C5b-9 deposition on the endothelium is a feature of microthrombotic diseases, which exacerbates endothelial damage [166,167]. Therefore, complement-mediated hyperinflammation and thrombosis by SARS-CoV-2 infection may underlie endothelial damage and subsequent microvascular clinical manifestations in severe COVID-19 [161,166,167]. This offers promise for development of complement-suppressing therapeutics aimed at decreasing hyperinflammation in severe COVID-19.

#### 6.2.2. Hypercoagulation and Hypofibrinolysis

**Figure 5:**
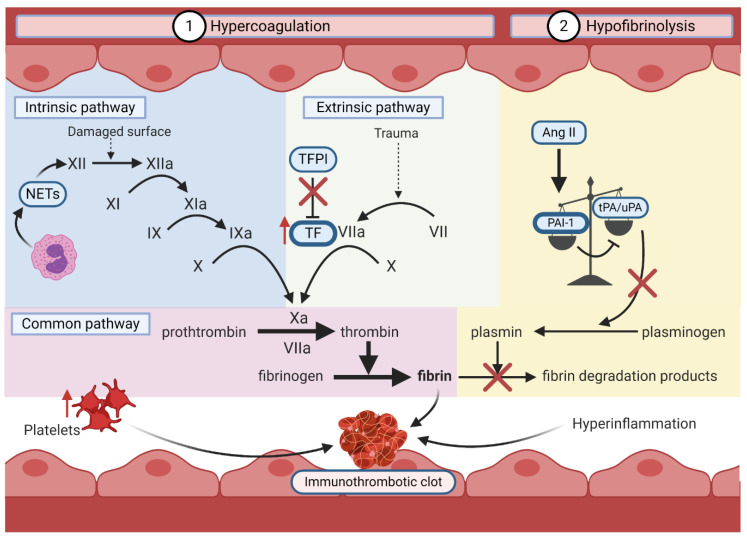
Endothelial dysfunction leads to immunothrombosis by inducing a pro-thrombotic state, hyperinflammation, and increasing platelets. Deleterious thrombosis is mediated by hypercoagulation and hypofibrinolysis. (1) The intrinsic coagulation pathway is stimulated by vascular endothelial damage and NET formation by PMNs. The extrinsic pathway is stimulated by COVID-19-mediated TF upregulation and TFPI inhibition. These pathways converge on the common pathway, promoting hypercoagulation through excessive fibrin formation. (2) Fibrinolysis is inhibited by the Ang II-mediated increased PAI-1:tPA/uPA ratio. Together, accumulation of fibrin, platelets, and hyperinflammation due to endothelial dysfunction contribute to the formation of immunothrombotic clots.

In COVID-19, the induction of a prothrombotic state is mediated by hypercoagulation and hypofibrinolysis (Figure 5). Direct or indirect endothelial dysfunction by SARS-CoV-2 orchestrates hypercoagulation by activating the intrinsic and extrinsic coagulation pathways through three major mechanisms.

First, loss of endothelium integrity increases the exposure of the thrombogenic basement membrane to the vasculature, which activates the intrinsic coagulation pathway. This allows the conversion of coagulation factor XII to its activated form (FXIIa). The activation cascade continues until the formation of a crosslinked fibrin clot [168]. The upregulation of polymorphonuclear leukocytes (PMNs) likely activates the intrinsic pathway in COVID-19 by releasing FXII-activating neutrophil extracellular traps [97,169]. Second, SARS-CoV-2-mediated endothelial damage exposes underlying tissue factor (TF) to coagulation factors in the blood [170]. This enables TF to activate FVII, also promoting the formation of a fibrin clot [170]. In hyperinflammation, PMNs and C3a/C5a increase endothelial TF exposure, while platelets and Ang II induce TF expression [119,162,170,171]. Finally, tissue factor pathway inhibitor (TFPI), an endogenous inhibitor of TF, is inhibited by pro-inflammatory cytokines, preventing homeostatic inhibition of the extrinsic coagulation pathway [170,171,172]. 

As previously described, induction of the RAAS by SARS-CoV-2-mediated ACE2 downregulation and/or shedding promotes hypofibrinolysis [97]. Hypercoagulation and hypofibrinolysis simultaneously foster a pro-thrombotic state, which with the addition of immune effectors and platelets, forms a highly pathogenic immunothrombotic clot in COVID-19 [152,153]. 

### 6.3. Impaired Antiviral Response

Recent evidences indicates that COVID-19 is associated with CD4+ and CD8+ T-cell lymphopenia [155,173,174,175,176]. These studies also show that decreased CD4+ and CD8+ cell counts correlate with worse prognosis and increased disease severity [155,173,174,175,176]. Since these cells express ACE2, lymphocyte deficiency may occur as a result of direct SARS-CoV-2 infection [177]. Supporting this claim is a study by Davanzo et al. [178], which presents evidence of SARS-CoV-2 infection in human primary CD4+ T-cells from COVID-19 patients that is associated with disease severity. 

Endothelial damage-mediated hyperinflammation in the context of SARS-CoV-2 infection is a known contributor to lymphopenia, as TNF-α and IL-6 have been shown to induce lymphocyte deficiency [175,179]. CD4+ T-cell lymphopenia may blunt the adaptive immune response to SARS-CoV-2, and even further exacerbate hyperinflammation through impaired downregulation of inflammatory mediators [16,97,174]. 

The adaptive immune response is also likely directly dysregulated by COVID-19-mediated endothelial damage. Vascular endothelial cells are facultative antigen presenting cells that have been shown to promote the killing of influenza- and vesicular stomatitis virus-infected cells by presenting viral peptides to CD8+ T-cells [180]. Endothelial damage and dysfunction would prevent endothelial cells from activating lymphocytes [180], but the degree to which this might occur with SARS-CoV-2 remains unclear. 

There is increasing interest in the involvement of monocytes and monocyte-derived macrophages in COVID-19 pathophysiology. Whether by virus-induced apoptosis in endothelial cells or by endothelial dysfunction caused by immunothrombosis, the damaged endothelium can secrete monocytes chemoattractants (CCL2, CCL7) [181]. Recruitment of pro-inflammatory blood monocytes and pro-inflammatory monocyte-derived macrophages in the lungs in COVID-19 has been observed in several studies [182,183,184]. In particular, the levels of pro-inflammatory CD14+CD16+ monocytes were elevated in the lungs of 28 hospitalized COVID-19 patients relative to healthy controls [184], which was corroborated by a study that found increased CD14+CD16+ monocytes with high expression of IL-6 in the peripheral blood of 33 COVID-19 patients [182]. IL-6 production by CD16+ monocytes exacerbates hyperinflammation in severe COVID-19 and is associated with lung pathology [182,185]. An observational study of 34 COVID-19 patients by Zhang et al. [184] found an unusual heterogeneous monocyte response with increased intracellular staining for pro-inflammatory IL-6 as well as anti-inflammatory IL-10. Simultaneous elevation of cytokines involved in type 1 (IL-6) and type 2 (IL-10) immune responses may reflect a multiphasic monocyte/macrophage response against SARS-CoV-2 infection [185]. By examining temporal and spatial characteristics of monocytes in African green monkey and rhesus macaque animal models with COVID-19, Fahlberg et al. [185] characterized an initial acute hyperinflammatory state with increased frequency of CD16+ monocytes in the lungs, followed by a gradual switch to a type 2 response, and corresponding increase in either anti-inflammatory IL-10 associated with disease resolution, or IL-6, which is associated with increased disease severity [185].

Interestingly, monocytes express ACE2 [184] and are permissive for SARS-CoV-1 [186], suggesting that they may also be susceptible to SARS-CoV-2 infection. To date, only abortive infection of SARS-CoV-2 has been described in monocytes and monocyte-derived macrophages [187,188]. Non-productive monocyte/macrophage infection by SARS-CoV-2 is also associated with production of immunoregulatory cytokines (IL-6, IL-10, IFN-α, IFN-β, IL-1) [187,188], which were shown to induce infected cell apoptosis via IFN-α/β receptor engagement [188]. It is therefore possible that abortive infection of monocytes/macrophages impairs the antiviral response against SARS-CoV-2 and contributes to disease progression [187,188]. Finally, a study of 69 COVID-19 patients found that CD4 expression on monocytes was decreased in the 16 patients with severe COVID-19 [189]. Since decreased CD4 on monocytes correlated with increased disease severity in this study, patients with severe COVID-19 may be unable to mount the required CD4-mediated activation of monocyte for viral clearance [189]. Together, these results support the possibility of aberrant monocyte and monocyte-derived macrophage responses in COVID-19, although the underlying immunopathogenesis requires further investigation.

## 7. Therapeutic Strategies for SARS-CoV-2-Mediated Endothelial Dysfunction

To date, there are five therapeutics approved by the US Food and Drug Administration (FDA) for emergency use in COVID-19 patients [190]. Amongst these, the NIH supports the use of antiviral nucleotide analogue drug remdesivir [29,30,31] and dexamethasone, a corticosteroid shown to reduce mortality in hospitalized patients receiving invasive ventilation or non-invasive oxygen delivery [29,34]. However, recent results from the randomized World Health Organization Solidarity Trials in 11,330 inpatients from 405 hospitals in 30 countries found that remdesivir had little or no effect on hospitalized COVID-19 patient mortality, initiation of ventilation, and duration of hospital stay [33]. Ongoing basic science research and clinical trials are investigating therapies that target COVID-19-related endothelial dysfunction.

### 7.1. Limiting SARS-CoV-2 Entry in Endothelial Cells

SARS-CoV-2 entry is mediated by the interaction between the viral S-protein and cellular receptors ACE2 and TMPRSS2. A research group developed a lipopeptide derived from a pan-coronavirus fusion inhibitor peptide (EK1C4) that reportedly inhibits S-protein-mediated membrane fusion in vitro [191]. Another approach to disrupt the interaction between the S-protein and ACE2 is by administration of human recombinant soluble ACE2 in different engineered cell types including blood vessels organoids (hrsACE2). hrsACE2 has recently been reported to reduce SARS-CoV-2 in vitro [141,192], and in a case report [142], likely by blocking the association of the S-protein with membrane-bound ACE2 [41]. The potential for hrsACE2 to block viral entry and decrease viral replication is being assessed in a European clinical trial (Clinical Trial NCT04335136). 

Neutralizing antibodies against the viral S protein are being developed for treatment of mild to moderate COVID-19. REGN-COV2, a cocktail of two potent neutralizing anti-S antibodies, has recently emerged as a promising prophylactic and therapeutic shown to reduces viral load in the airways of rhesus macaques and golden hamsters [193]. The monoclonal antibody bamlanivimab (LY-CoV555) received emergency use authorization (EUA) from the FDA for treatment of mild to moderate COVID-19 [190] after being shown to reduce COVID-19 hospitalization and emergency room visits in 467 non-hospitalized patients at high risk for disease progression (interim analysis Clinical Trial NCT04427501) [194]. Shortly thereafter, the neutralizing antibodies casirivimab (REGN10933) and imdevimab (REGN10987) also received FDA EUA for treatment of mild to moderate COVID-19 at high risk for disease progression [190]. When co-administered, these drugs reduced hospitalization and emergency room visits in a randomized clinical trial of 799 non-hospitalized mild to moderate COVID-19 patients (interim analysis of Clinical Trial NCT04425629) [195]. The final results of casirivimab and imdevimab combination therapy in the randomized phase 1/2/3 trials in COVID-19 patients (Clinical Trials NCT04425629, NCT04426695) had not been released at the time of writing. 

### 7.2. RAAS Inhibition

The RAAS pathway is deleteriously activated as a result of ACE2 downregulation on endothelial cells following SARS-CoV-2 entry and/or ACE shedding. Inhibition of the RAAS pathway using ACE2 inhibitors (ACEi) and angiotensin receptor blockers (ARBs) has thus been proposed as a therapeutic strategy [112,196].

Inpatient use of ACEi/ARBs is associated with improved clinical outcomes and decreased mortality in COVID-19 patients [112,196,197]. ACEi administration reportedly lowers PAI-1 and increases tPA, while ARBs have variable effects on PAI-1 and no apparent effect on tPA levels [97]. Nevertheless, blocking the AT1R was shown to attenuate acute lung injury and pulmonary edema in Spike-Fc-treated mice [110]. However, a study that retrospectively investigated the relationship between RAAS inhibitors and COVID-19 in-hospital mortality of 4069 patients found no association between ACEi/ARB treatment and in-hospital mortality [198]. Other clinical trials are underway to examine the effects of ACEi/ARBs on COVID-19 severity (Clinical Trial NCT04364984, NCT04364893, NCT04353596). Several other randomized clinical trials investigating the use of the ARB losartan for hypertension in COVID-19 are also being conducted (Clinical Trial NCT04311177, NCT04394117, NCT04367883). Conclusions from these clinical studies should highlight the need to identify optimal therapeutic targets of the RAAS.

### 7.3. Immunomodulation

SARS-CoV-2 suppresses induction of the innate antiviral response in early stages of infection by inhibiting the type I IFN response [155,156]. It has been demonstrated that early administration of type I IFNs inhibits viral replication in vitro by partially restoring innate immune response to SARS-CoV-2 infection [156]. A randomized clinical trial of 80 patients with moderate COVID-19 and pneumonia found that aerosol inhalation of IFN-ĸ (a type I IFN) combined with TFF2 anti-inflammatory polypeptide led to faster SARS-CoV-2 clearance and facilitated clinical resolution of pneumonia [199]. A phase 2 clinical trial assessing the efficacy of IFN-β (a type I IFN) treatment in 127 COVID-19 patients found that IFN-β in combination with lopinavir-ritonavir and ribavirin shortened viral shedding time and hospital stay [200]. There is another clinical trial underway to assess the efficacy of IFN-β in combination with lopinavir/ritonavir (Clinical Trial NCT04315948), and several others for IFN-β in combination with clofazimine (Clinical Trial NCT04465695) and ribavirin (Clinical Trial NCT04494399) for the treatment of COVID-19.

As endothelial dysfunction progresses, inflammation is amplified by the release of pro-inflammatory cytokines by immune and endothelial cells [22,155,171]. Tocilizumab is an antibody that blunts IL-6-mediated hyperinflammation by binding to IL-6 receptor [17]. Several studies have found that Tocilizumab reduces mortality and improves clinical outcomes in patients with severe COVID-19 [201,202]. However, a randomized clinical trial of 243 moderately ill COVID-19 patients found that Tocilizumab did not effectively prevent intubation or death in the treatment group [203]. Several ongoing clinical trials investigating the therapeutic effect and tolerance of Tocilizumab should further elaborate whether this treatment significantly impacts COVID-19 presentation. The Bruton tyrosine kinase inhibitor acalabrutinib also mitigates hyperinflammation by regulation of macrophage signaling and activation [204] and is currently being investigated for efficacy in clinical trials for COVID-19 (Clinical Trial NCT04647669, NCT04380688).

Activation of the Janus kinase-Signal Transducer and Activator of Transcription (JAK-STAT) signaling pathway by cytokines (especially IL-6) released in SARS-CoV-2-mediated hyperinflammatory processes has been proposed to contribute to viral entry and exacerbation of inflammation [205,206]. Richardson et al. [206] demonstrated that the JAK inhibitor baricitinib prevented viral entry by blocking regulators of endocytosis in the JAK-STAT pathway, which was followed by a phase 3 randomized clinical trial of 1033 moderate to severe COVID-19 patients that found administration of baricitinib with remdesivir decreased recovery time [207]. Subsequently, baricitinib in combination with remdesivir received EUA from the FDA for use in hospitalized COVID-19 patients requiring oxygenation or ventilation [208].

Ruxolitinib is another JAK1/2 inhibitor that has shown promise to limit hyperinflammation in COVID-19 in a pilot case series of 105 COVID-19 patients [209]. Another study of 43 COVID-19 patients found that ruxolitinib administration had clinical benefits including faster recovery from lymphopenia, although no statistical significance was reported [210]. We expect results from current clinical studies to elucidate safety and efficacy of ruxolitinib in COVID-19 patients (Clinical trials NCT04377620, NCT04334044, NCT04477993).

### 7.4. Inhibition of Thrombosis

Severe COVID-19 is associated with thrombotic complications due to hypercoagulation and hypofibrinolysis [97,153]. These processes have therefore been designated as therapeutic targets for COVID-19. Low-molecular-weight heparin (LMWH) is an anticoagulant medication commonly used to treat venous thromboembolism and inflammation [17,211]. LMWH is as an inhibitor of the endothelial glycocalyx-degrading enzyme heparanase, which is well known to exacerbate vascular leakage and inflammation, and may be involved in the development of ARDS in COVID-19 [212]. A study of 48 hospitalized COVID-19 patients found evidence of endothelial barrier destruction and increased plasma heparanase associated with disease severity [212]. However, anticoagulation therapy with LMWH was associated with a better prognosis in severe COVID-19 patients [211,212].

Interestingly, heparin reportedly also inhibits SARS-CoV-2 infection directly by competing for viral S-protein binding [66,213]. Ongoing clinical studies should clarify the potential of LMWH to treat thrombotic complications and decrease possible SARS-CoV-2 endothelial infection in the context of COVID-19 (Clinical Trial NCT04492254, NCT04542408, NCT04393805, NCT04584580, NCT04373707).

Fibrinolytic therapies are also being considered to address thrombotic complications of COVID-19. After viral infection, the ratio of PAI-1 to tPA/uPA is increased, resulting in hypofibrinolysis [97,104,119,120]. Administrating tPA may restore thrombotic homeostasis by increasing fibrinolysis [97]. Several clinical trials are investigating the role of tPA in ARDS and thrombotic complications of COVID-19 (Clinical Trial NCT04453371, NCT04357730, NCT04356833). 

## 8. Conclusions

This paper provides a summary of the proposed relationship between COVID-19 and endothelium dysregulation. Although there remain controversies regarding the direct infection of endothelial cells by SARS-CoV-2, it appears endothelium dysfunction is responsible for microvascular complications in severe COVID-19. SARS-CoV-2 may cause endothelium dysfunction directly by downregulating cellular ACE2 or indirectly by impairing normal pericyte–endothelial cross-talk, potentiating immunothrombosis, and impairing the antiviral response. Endothelial dysfunction in patients with comorbidities (hypertension, diabetes, obesity) combined with SARS-CoV-2-induced endothelial dysfunction likely contribute to deleterious pulmonary and extra-pulmonary complications in COVID-19. Ongoing and future pre-clinical and clinical studies investigating the role of the endothelium in SARS-CoV-2 pathogenesis will advance the rational development of therapeutics alleviating pulmonary and extra-pulmonary vascular complications in COVID-19. 

## Supplementary Materials

The following are available online at https://www.mdpi.com/1999-4915/13/1/29/s1, Supplementary Material S1: Literature search strategy.
