# Supplementary material for: Endothelium Infection and Dysregulation by SARS-CoV-2: Evidence and Caveats in COVID-19
Source: Viruses. 2020 Dec 26;13(1):29. doi: 10.3390/v13010029 (PMC7823949; doi:10.3390/v13010029)
Supplement: Supplementary file 1 [file viruses-13-00029-s001.pdf]

Endothelium infection and dysregulation by SARS-CoV-2: evidence and caveats in COVID-19

The following databases were searched:
- PubMed
- Ovid MEDLINE
- Google Scholar
- bioRxiv and medRxiv
- ClinicalTrials.gov

Date last search was run: December 14, 2020
Years covered by search: All years available in each database were included in the search
Language restriction: Only articles published in English were included in this review
Document restriction: No document types restriction

PubMed Search Strategy

No	Query	Results
1	"SARS-CoV-2" [Title/Abstract] OR "COVID-19" [Title/Abstract] OR "2019-nCoV" [Title/Abstract]	76, 854
2	"cell" [Title/Abstract] OR "endothelium" [Title/Abstract] OR "endothelial" [Title/Abstract]	3, 666, 410
3	Combined 1 and 2: ("SARS-CoV-2" [Title/Abstract] OR "COVID-19" [Title/Abstract] OR "2019-nCoV" [Title/Abstract]) AND ("cell" [Title/Abstract] OR "endothelium" [Title/Abstract] OR "endothelial" [Title/Abstract])	3, 998

Ovid MEDLINE search strategy

No	Query	Results
1	(COVID-19 or SARS-CoV-2 or 2019-nCoV).mp. [mp=title, abstract, original title, name of substance word, subject heading word, floating sub-heading word, keyword heading word, organism supplementary concept word, protocol supplementary concept word, rare disease supplementary concept word, unique identifier, synonyms]	81, 612
2	(endothelium or endothelial or cell).mp.	4, 676, 677
3	Combined 1 and 2: ((COVID-19 or SARS-CoV-2 or 2019-nCoV) and (endothelium or endothelial or cell)).mp.	4, 492

Google Scholar search strategy

No	Query	Results
1	"COVID 19" OR "SARS CoV 2" OR "2019 nCoV"	About 19, 000
2	“endothelium” OR “endothelial” OR “cell”	About 7, 470, 000
3	“endothelium” "COVID 19" OR "SARS CoV 2" OR "2019 nCoV"	13, 200
4	“endothelial” "COVID 19" OR "SARS CoV 2" OR "2019 nCoV"	16, 300

bioRxiv and medRxiv search strategy

*includes articles in bioRxiv only, medRxiv only, and bioRxiv and medRxiv

No	Query	Results
1	for title "SARS-CoV-2, COVID-19, 2019-nCoV" (match any words)	10, 202
2	for abstract or title "endothelium, endothelial, cell" (match any words)	1, 082
3	Combined 1 and 2:<br>for title "SARS-CoV-2, COVID-19, 2019-nCoV" (match any words) and abstract or title "endothelium, endothelial, cell" (match all words)	11, 209

ClinicalTrials.gov search strategy

No	Query	Results
1	Initial search was (“endothelium | Covid 19”). Database also automatically searched for (“Endothelial”, “COVID”, and “SARS-CoV-2”).	87

These search strategies were used to identify literature relevant to endothelial infection and dysfunction in COVID-19. Using this search strategy, subsequent searches were also made by adding specific terms related to endothelial involvement in COVID-19 and related to SARS, SARS-CoV-2, and SARS-CoV.

For example, **PubMed search for topics related to COVID-19 and endothelium:**

**Query**	**Results**
("SARS-CoV-2" [Title/Abstract] OR "COVID-19" [Title/Abstract] OR "2019-nCoV" [Title/Abstract]) AND ("cell" [Title/Abstract] OR "endothelium" [Title/Abstract] OR "endothelial" [Title/Abstract])	3, 998
(((SARS-CoV-2[Title/Abstract]) OR (COVID-19[Title/Abstract]) OR (2019-nCoV[Title/Abstract])) AND ((cell[Title/Abstract]) OR (endothelium[Title/Abstract]) OR (endothelial[Title/Abstract]))) AND (endotheliitis[Title/Abstract])	36
(((SARS-CoV-2[Title/Abstract]) OR (COVID-19[Title/Abstract]) OR (2019-nCoV[Title/Abstract])) AND ((cell[Title/Abstract]) OR (endothelium[Title/Abstract]) OR (endothelial[Title/Abstract]))) AND (vascular[Title/Abstract])	275
(((SARS-CoV-2[Title/Abstract]) OR (COVID-19[Title/Abstract]) OR (2019-nCoV[Title/Abstract])) AND ((cell[Title/Abstract]) OR (endothelium[Title/Abstract]) OR (endothelial[Title/Abstract]))) AND (thrombosis[Title/Abstract])	207
(((SARS-CoV-2[Title/Abstract]) OR (COVID-19[Title/Abstract]) OR (2019-nCoV[Title/Abstract])) AND ((cell[Title/Abstract]) OR (endothelium[Title/Abstract]) OR (endothelial[Title/Abstract]))) AND (immunothrombosis[Title/Abstract])	14
(((SARS-CoV-2[Title/Abstract]) OR (COVID-19[Title/Abstract]) OR (2019-nCoV[Title/Abstract])) AND ((cell[Title/Abstract]) OR (endothelium[Title/Abstract]) OR (endothelial[Title/Abstract]))) AND (complement[Title/Abstract])	78
(((SARS-CoV-2[Title/Abstract]) OR (COVID-19[Title/Abstract]) OR (2019-nCoV[Title/Abstract])) AND ((cell[Title/Abstract]) OR (endothelium[Title/Abstract]) OR (endothelial[Title/Abstract]))) AND (coagulation[Title/Abstract])	187
(((SARS-CoV-2[Title/Abstract]) OR (COVID-19[Title/Abstract]) OR (2019-nCoV[Title/Abstract])) AND ((cell[Title/Abstract]) OR (endothelium[Title/Abstract]) OR (endothelial[Title/Abstract]))) AND (fibrinolysis[Title/Abstract])	13
(((SARS-CoV-2[Title/Abstract]) OR (COVID-19[Title/Abstract]) OR (2019-nCoV[Title/Abstract])) AND ((cell[Title/Abstract]) OR (endothelium[Title/Abstract]) OR (endothelial[Title/Abstract]))) AND (monocytes[Title/Abstract])	86
(((SARS-CoV-2[Title/Abstract]) OR (COVID-19[Title/Abstract]) OR (2019-nCoV[Title/Abstract])) AND ((cell[Title/Abstract]) OR (endothelium[Title/Abstract]) OR (endothelial[Title/Abstract]))) AND (RAAS[Title/Abstract])	35
(((SARS-CoV-2[Title/Abstract]) OR (COVID-19[Title/Abstract]) OR (2019-nCoV[Title/Abstract])) AND ((cell[Title/Abstract]) OR	867

(endothelium[Title/Abstract]) OR (endothelial[Title/Abstract]))) AND (ACE2[Title/Abstract])	
(((SARS-CoV-2[Title/Abstract]) OR (COVID-19[Title/Abstract]) OR (2019-nCoV[Title/Abstract])) AND ((cell[Title/Abstract]) OR (endothelium[Title/Abstract]) OR (endothelial[Title/Abstract]))) AND (bradykinin[Title/Abstract])	12
(((SARS-CoV-2[Title/Abstract]) OR (COVID-19[Title/Abstract]) OR (2019-nCoV[Title/Abstract])) AND ((cell[Title/Abstract]) OR (endothelium[Title/Abstract]) OR (endothelial[Title/Abstract]))) AND (ADAM17[Title/Abstract])	16
(((SARS-CoV-2[Title/Abstract]) OR (COVID-19[Title/Abstract]) OR (2019-nCoV[Title/Abstract])) AND ((cell[Title/Abstract]) OR (endothelium[Title/Abstract]) OR (endothelial[Title/Abstract]))) AND (pericyte[Title/Abstract])	3
(((SARS-CoV-2[Title/Abstract]) OR (COVID-19[Title/Abstract]) OR (2019-nCoV[Title/Abstract])) AND ((cell[Title/Abstract]) OR (endothelium[Title/Abstract]) OR (endothelial[Title/Abstract]))) AND (therapeutics[Title/Abstract])	193
